# Oleogels with Birch Bark Dry Extract: Extract Saving Formulations through Gelation Enhancing Additives

**DOI:** 10.3390/pharmaceutics12020184

**Published:** 2020-02-21

**Authors:** Kashif Ahmad Ghaffar, Rolf Daniels

**Affiliations:** Department of Pharmaceutical Technology, Eberhard Karls University, Auf der Morgenstelle 8, 72076 Tuebingen, Germany; kashif.ghaffar@ymail.com

**Keywords:** birch bark extract, oleogels, hydrogen bonding, triterpene, rheology, gel strength

## Abstract

Triterpenes from the outer bark of birch have many beneficial biological and pharmacological activities. In particular, its wound healing efficacy is of paramount importance. Apart from that, particles of a birch bark dry extract aggregate into a three dimensional network when they are dispersed in lipids yielding a semi-solid oleogel. However, gel formation requires high amounts of the extract, which then acts at once as the active ingredient and the gelling agent. Infrared spectra of the respective mixtures proved that hydrogen bonds play a crucial role in the formation of the gel network. Dicarboxylic acids had almost no effect on gel strength. Monoalcohols increased the firmness of the oleogel with a decreasing effect from methanol > ethanol > butanol > octanol. All tested terminal diols increased the gel strength whereas vicinal diols affected the gel strength negatively. The effect was highly dependent on their concentration. The different effects of the diols are linked to their structure and polarity. The most pronounced enhancement of gelation was found for 1,6-hexanediol, which reduced the amount of triterpene extract (TE), which is necessary for the formation of an oleogel by a factor of 10.

## 1. Introduction

**Figure 1:**
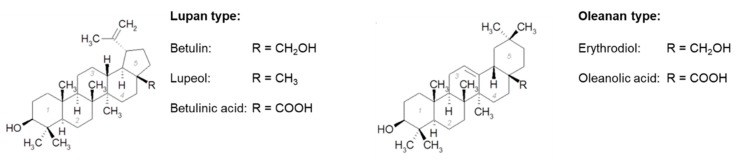
Chemical structure of triterpenoids with Lupan and Oleanan type skeleton.

It is well known, that triterpenoids, widespread secondary plant metabolites, have many beneficial biological and pharmacological activities [1,2]. Triterpenes from birch bark are of exceptional interest. The following triterpenes are the most common in the outer bark of the white birch: betulin, lupeol, betulinic acid, oleanolic acid, and erythrodiol (Figure 1) [3,4].

There are different ways described in the literature to extract and isolate these triterpenes, such as extraction of triterpenoids with organic solvents like n-heptane [4,5] or ethanol [6]. Other methods used include supercritical fluid technology [3], ultrasonic-assisted extraction [7], and sublimation [8].

Triterpenes have antiviral, antibacterial, and antitumoral activities [9,10]. Moreover, a triterpene extract from the outer bark of birch was shown to enhance wound healing ex vivo as well as in vivo in humans [11,12,13]. In January 2016 an oleogel consisting of solely sunflower oil and birch bark extract got European marketing authorization for the treatment of partial-thickness wounds [11]. In this formulation, the triterpene extract functions as the active substance as well as an excipient. In such binary mixtures, the extract forms a three-dimensional gel network and immobilizes the liquid oil [12]. A similar aggregation of triterpenes through hydrogen bonds was also shown in organic solvents [13,14]. The aggregation of a substance and the resulting gelling effect is also described for so called organogels, where different classes of gelling agents such as glycerol ester, fatty acid, fatty alcohols, or sterols are used [15]. These are so called low-molecular-weight-gelators, and 12-hydroxystearic acid was one of the first substances investigated intensively; it was shown that both its hydroxyl and carboxyl group play a crucial role during gelation [16]. These gelators are also of exceptional interest in the food industry as an alternative to saturated triglycerides for structuring oils, in which secondary valence bonds (e.g., hydrogen bonds) play a vital role [17]. It was also shown that specific substances are able to induce the interaction of the gelling agents and thus increase the gel strength of such organogels [18,19]. In the same direction, results with triterpene extract (TE) oleogels indicated that the gel strength is affected by a complex interplay of liquid–solid interaction as well as the interaction of suspended TE particles. Up to date it is not fully understood how this kind of gelation is exactly brought about and how additives affect it. However, there are clear hints that hydrogen bonds play an essential role in this process [12]. From a formulator’s point of view, the formation of a particulate gel network using the active ingredient might be problematic because it has to be added in comparably large amounts. Moreover, it is an inherent disadvantage that the concentration of the active ingredient and the gelator cannot be changed independently. Finally, the formation of a three-dimensional particulate network takes a lot of time because the rearrangement of the particles is a rather slow process.

Therefore, the aim of this study was to (1) find additives which are able to increase the gel strength and accelerate the gel formation and (2) further increase knowledge on the mechanism behind the formation of the particulate gel network. To this end, the strength of oleogels consisting of birch bark extract, sunflower oil, and several additives that might form H-bonds was studied by means of oscillation rheology. The interaction between TE particles and additives was characterized by IR spectroscopy. Furthermore, the effect of the additives on the solubility of the TE was analyzed.

## 2. Materials and Methods

### 2.1. Materials

The dry birch bark extract was a gift from Amryt AG (Niefern-Oeschelbronn, Germany). It was produced by enhanced organic solvent extraction using n-heptane [5]. The chemical and physical characterization of the extract is shown in Table 1.

1,2-Propanediol was obtained from Caesar & Loretz GmbH (Hilden, Germany). Methanol, ethanol, butanol, octanol, ethylene glycol, 1,3-butanediol, 1,4-butanediol, 1,5-pentanediol, 1,2-hexanediol, 1,6-hexanediol, 2,5-hexanediol, 1,9-nonanediol, adipic acid, and azealic acid were purchased from Sigma Aldrich (Taufkirchen, Germany) with a purity >95%. Succinic acid was from Merck KGaA (Darmstadt, Germany) and 1,2-pentanediol was from BASF (Zurich, Switzerland).

### 2.2. Gel Preparation

The dry triterpene extract was sieved through a number 500 sieve before further use. Thereafter, an 8% stock oleogel was produced by homogenizing the extract for 2 min at 8500 rpm in sunflower oil by means of an Ultra Turrax (Ika Labortechnik, Staufen, Germany). The additives were added to the stock oleogel in a predetermined amount and the final formulation was again homogenized for 1 min at 8500 rpm. Oleogels were analyzed 24 h after preparation.

### 2.3. Rheology

Rheology of the oleogels was measured in oscillation mode using a Gemini 150 (Bohlin Instruments, Pforzheim, Germany) equipped with a 25 mm plate/plate geometry using a gap of 1 mm. After a pre-shear phase of 40 s and 5 min recovery time, an amplitude sweep was performed with a logarithmic ramp, deformation rate of 0.01–1000%, and a frequency of 1 s^−1^. The gel strength of the oleogels was expressed as the mean of the storage modulus G’ in the linear viscoelastic range. In general, gel strength of all oleogels was measured 24 h after preparation in order to allow formation of the gel network after intense shearing during the manufacturing process.

### 2.4. ATR–Infrared Spectroscopy

The samples were analyzed with a Spectrum One FTIR-Spectrometer (Perkin Elmer, Rodgau, Germany). They were placed on the surface of an optical fiber and spectra were taken with a wave number ranging from 4000 cm^−1^ to 670 cm^−1^. In total, 31 spectra were cumulated and evaluated using the software Spectrum v. 3.01 (Perkin Elmer, Rodgau, Germany).

### 2.5. Particle Size Measuring

The particle size distribution of the oleogels was measured through laser diffraction using a Sympatec Helos (Sympatec GmbH, Clausthal-Zellerfeld, Germany), with a lens focal length of 100 mm and measuring range of 0.9–175 µm. For these measurements oleogels were produced with light liquid paraffin and an extract concentration of 8%. After diluting the sample in a ratio of 1:10 with the oil phase, the sample was added in a cuvette where it was stirred in paraffin to get a homogeneous dispersion. Measurements were taken at an optical concentration of 25–30%. 

Laser diffraction results are expressed as ×_10_, ×_50_, and ×_90_ values based on a cumulative volume distribution. To ensure comparability between rheology and particle size measurements, respective oleogels for particle sizing were also stored at 25 °C.

## 3. Results and Discussion

### 3.1. Effect of Additives on Gel Strength

**Figure 2:**
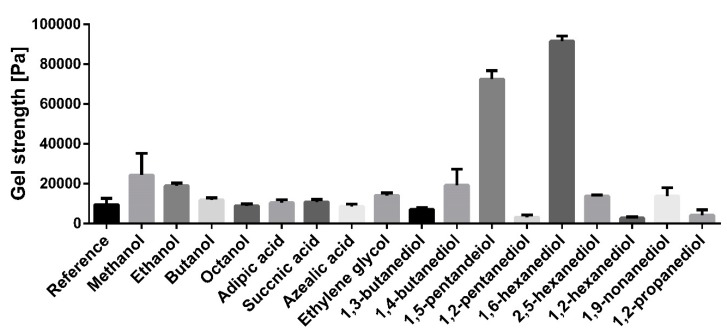
Gel strength of oleogels with sunflower oil, 8% triterpene extract (TE), and 2% additive; an oleogel without any additive serves as a reference; mean ± SD, *n* = 3.

Previous studies dealing with oleogel formation with TE particles hypothesized that the formation of the gel network is markedly assisted by dissolved bi-functional triterpene molecules, (e.g., betulin). In order to check this, the effect of several mono- and bi-functional additives was studied systematically. Figure 2 shows the effect of the different additives on the gel strength of an oleogel consisting of sunflower oil and 8% triterpene extract.

Most tested excipients with hydroxyl functional groups were able to increase gel strength. In the series of the mono-functional alcohols, the gel strength decreased with their chain length. The addition of methanol gave the highest increase in gel strength. None of the used dicarboxylic acids (adipic acid, succinic acid, azelaic acid) affected the gel strength, which is explained by the creation of cyclic dimers of the dicarboxylic acids [20,21]. The additives prefer interacting with each other instead of the extract particles. All diols with terminal hydroxyl groups increased the gel strength. The effect was dependent on the alkyl chain length of the diols and showed maximum values with 1,6-hexanediol, leading to a 9-fold increase in viscosity when 2% of this substance were added. The gel strength decreased again when the chain length further increased. Diols with vicinal hydroxyl groups (e.g., 1,2-hexanediol), affected the gel strength negatively. Again, the effect was dependent on the chain length. Accordingly, the most prominent reduction in gel strength was seen with 1,2-hexanediol.

### 3.2. IR-Measurements

**Figure 3:**
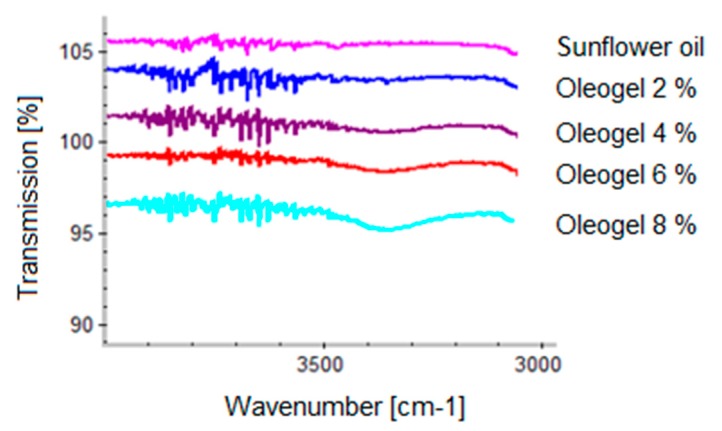
IR spectra of oleogels in sunflower oil with increasing TE concentration.

It is well known that gel is formed through hydrogen bonds between extract particles, which is shown by IR spectroscopy [12]. Figure 3 shows IR spectra of plain TE oleogels prepared with increasing TE concentrations.

**Figure 4:**
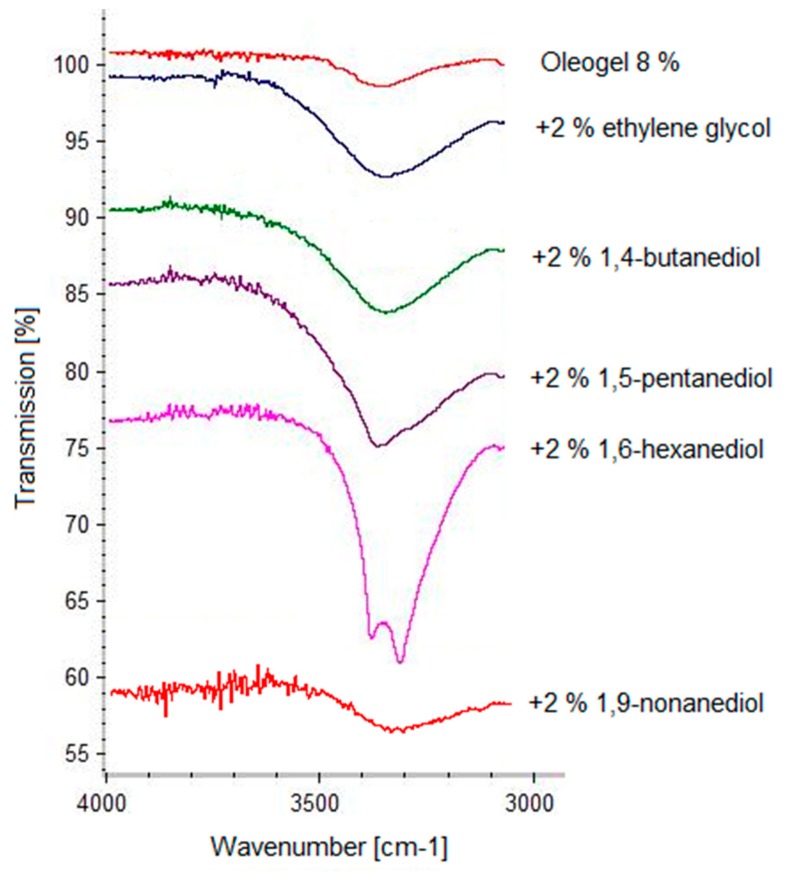
IR spectra of oleogels (8% TE) in sunflower oil with terminal diols (2%).

Free hydroxyl groups usually show an absorption band at 3700 cm^−1^. If the hydroxyl groups are involved in hydrogen bonds the absorption band is shifted to the 3500–3200 cm^−1^ wavenumber range. In Figure 3 it is clearly seen that the absorption band at 3300 cm^−1^ increases with increasing TE concentration. This indicates an increasing number of hydrogen bonds, which is in line with an increasing gel strength [12]. Diols have been shown to markedly affect gel strength in a positive or negative manner. IR spectra were taken to further elucidate the behavior of the diols. Figure 4 shows the IR spectra of a plain 8% TE oleogel in comparison to those measured after the addition of several diols with terminal hydroxyl groups, which enhanced the gel strength.

**Figure 5:**
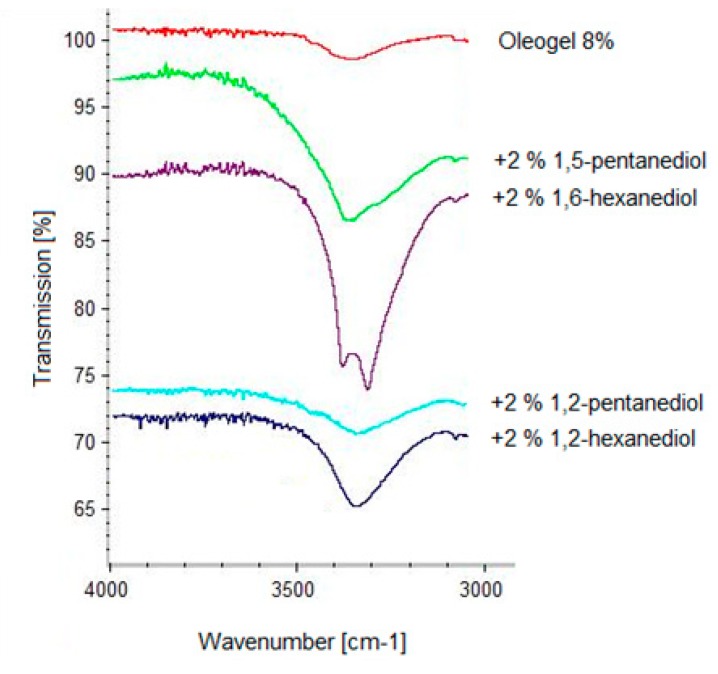
IR spectra of oleogels (8% TE) in sunflower oil with vicinal diols and their corresponding terminal diols (2%).

As expected, the IR spectra of the oleogels with the gel enhancing additives showed a fortified broad absorption band at 3400 cm^−1^, indicating that these diols are able to interact intensely with the extract particles via hydrogen bonds. The characteristic absorption band increased in the series from ethylene glycol to 1,6-hexanediol, which corresponds to an increasing firmness of the oleogel. The intense interaction of 1,6-hexanediol was accompanied by a splitting of the absorption band at 3400 cm^−1^ probably due to interactions between adjacent molecules. As demonstrated by 1,9-nonanediol, a further increase in the chain length of the terminal diol resulted in a reduction of both the absorption band at 3400 cm^−1^ as well as the gel strength. Figure 5 shows IR spectra of a plain 8% TE oleogel and after the addition of pentanediols and hexanediols with either terminal or vicinal hydroxyl groups.

**Figure 6:**
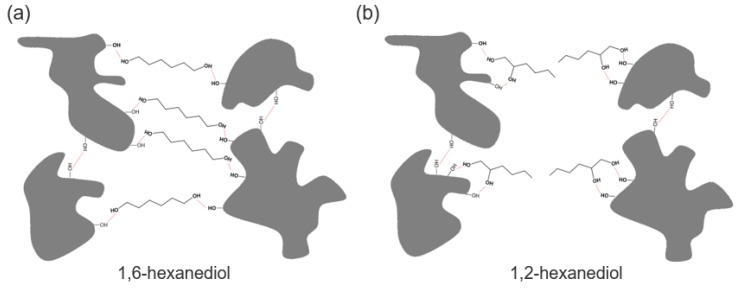
Schematic drawing of interaction between (**a**) 1,6-hexanediol and extract particle; (**b**) 1,2-hexanediol and extract particle (dimensions are not to scale).

Independent from the chain length of the diols, the absorption band at 3400 cm^−1^ is significantly smaller when adding vicinal diols instead of terminal diols. However, although a marked hydrogen bonding of the vicinal diols is still detectable, they do not increase the gel strength but rather reduce the firmness of the respective oleogels. As the absorption band of oleogels with vicinal diols is still larger compared to the plain oleogel, it could be concluded that the vicinal diols interact substantially with the superficial hydroxyl groups of the TE particles, but they are not able to link the TE particles together. To the contrary, they shield the superficial hydroxyl groups of the TE particles and hence prevent them from building a three dimensional particulate gel network as exemplary visualized in Figure 6.

Like the gel enhancing effect of the terminal diols, the gel weakening effect of the vicinal diols and the absorption band at 3400 cm^−1^ were more pronounced with increasing chain length of the diols.

### 3.3. Influence of the Enhancer Concentration on the Gel Strength

**Figure 7:**
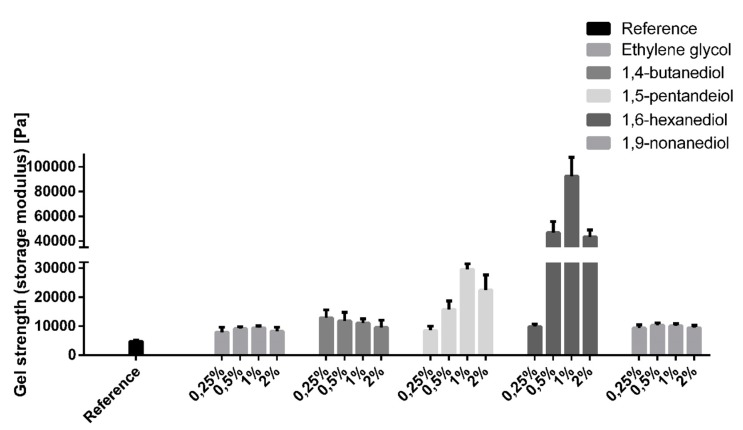
Gel strength of oleogels (8%) with terminal diols in a concentration range of 0.25–2%; an oleogel without any additive serves as a reference; mean ± SD, *n* = 3.

As the number of interacting superficial hydroxyl groups on the TE particles is limited it was of great interest if the effect of the additives on the gel strength depends on their concentration. To this end, oleogels were produced with terminal diols in the concentration range from 0.25–2% (corresponding to approx. 2–30 µmol m^−2^). As shown in Figure 7, the gelation enhancing effect of terminal diols was highly dependent on their concentration, with a maximum effect mostly in the range of 0.5–1%.

The firmness of the oleogels decreased again when the diol concentration exceeded the optimal range. However, this did not come with a decreasing absorption band at 3400 cm^−1^ (data not shown). Therefore, it is hypothesized that the surplus of diol forms hydrogen bonds with other diol molecules instead of the TE particles, leading to a less rigid three-dimensional network.

### 3.4. Particles Size of Oleogels

**Figure 8:**
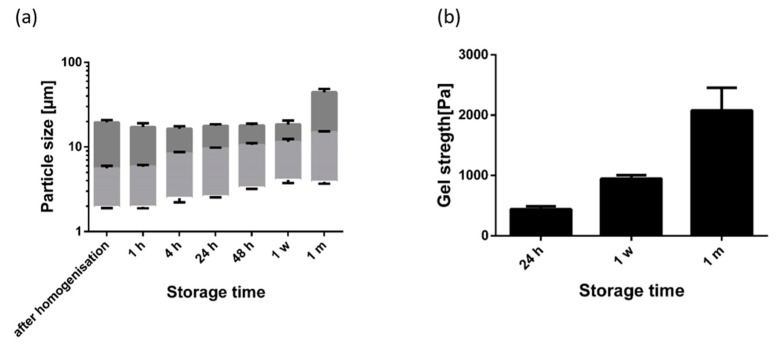
(**a**) Particle size distribution (×_10_, ×_50_, and ×_90_ values) and (**b**) gel strength of an oleogel (8% TE in paraffin) during one month of storage; mean ± SD, *n* = 3.

Fumed silica particles are comparable to gel oils by forming a three dimensional network [22]. The gelling mechanism is very similar compared to the triterpene oleogels. In oleogels using silica particles, the particles grow during storage because of further aggregation of the particles. This leads to a higher gel strength. We expected a similar particle growth with the TE oleogels. Moreover, 1,2-hexanediol was expected to have a negative effect, and 1,6-hexanediol to have a positive effect, on the growth of particle aggregates in line with the observed effect on gel strength. Figure 8 shows the particle size distribution and the gel strength of a plain oleogel during its one month storage at 25 °C.

As can be seen, laser diffraction revealed minimal particle size directly after homogenization. Due to aggregation, the measured particle size (×_10_ and ×_50_ value) subsequently increased steadily during storage. This aggregation of the particle is in line with measurements of the gel strength of the oleogel which also rises during storage. During storage, the ×_90_ values remained almost unaffected within the first week of storage and showed a significant increase after 1 month. This indicates that the interaction forces increased during storage to such an extent that the small shear forces during sample preparation were not sufficient to further disaggregate the larger aggregates completely.

**Figure 9:**
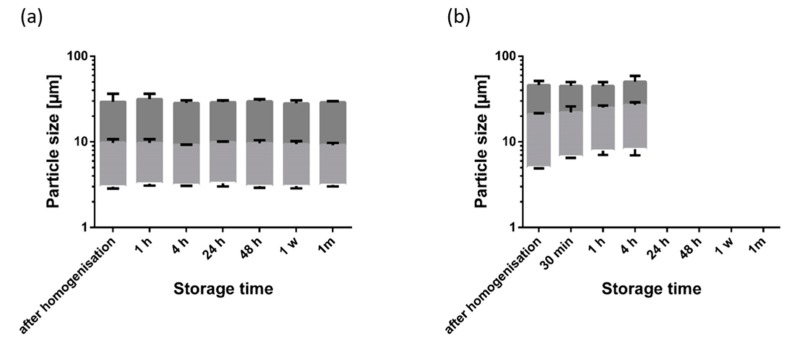
Particle size distribution of oleogels (8% TE in paraffin) during one month of storage with (**a**) 2% 1,2-hexanediol and (**b**) 2% 1,6-hexanediol; mean ± SD, *n* = 3.

This behavior changed substantially in the presence of 1,2-hexanediol and 1,6-hexanediol (Figure 9).

While 1,2-hexanediol inhibited particle aggregation during one month of storage, the measured particle size in the presence of 1,6-hexanediol was significantly larger directly after homogenization. Four hours after preparation, the particle size distribution was already comparable to the plain oleogel after one month of storage. The firmness of the oleogel with 1,6-hexanediol was too high to allow a particle size measurement after 4 h. Obviously, 1,6-hexandiol extensively promotes the interaction of TE particles.

**Figure 10:**
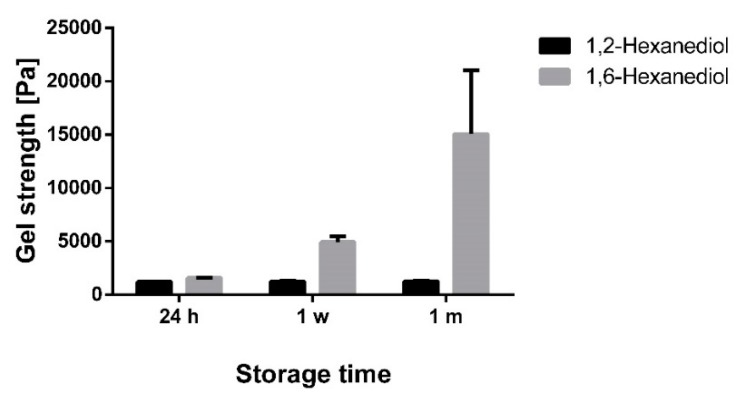
Gel strength of oleogels (8% TE in paraffin) during one month of storage with 2% 1,2-hexanediol and 1,6-hexanediol; mean ± SD, *n* = 3.

Again, the gel strength of the oleogels were in line with the results of the particle size measurements (Figure 10).

During one month of storage, the gel strength in the presence of 1,2-hexanediol remained almost constant at a low level due to insufficient interaction of the TE particles. In contrast, oleogels with 1,6-hexanediol showed a marked increase in the gel strength during one month of storage. At first sight, this behavior is comparable to plain oleogels, however, the absolute values of the gel strength are much higher in the presence of 1,6-hexanediol.

The mechanism behind these huge differences between 1,2-hexanediol and 1,6-hexanediol is already illustrated schematically in Figure 7. 1,6-Hexanediol with its terminal OH groups has the ability to act very effectively as a linker between the TE particles. Due to its much smaller size compared to the TE particles, 1,6-hexanediol is able to link the particles faster and more flexibly and thus more efficiently lead to a pronounced activation of the gel network. 1,2-Hexanediol with its vicinal OH groups has an amphiphile and adsorbs to the superficial functional groups of the TE particles. Consequently, the hydrocarbon chain is oriented to the continuous phase and prevents the formation of hydrogen bonds. This is comparable to the negative impact on the aggregation of silica particles dispersed in DMSO when the number of sites for hydrogen bonding of silanol groups was reduced by surface modification [22].

### 3.5. Extract Saving Effect of Additives

In the oleogels, TE represents the active component as well as the gel forming excipient. This brings about that higher amounts of TE are required than probably necessary for the intended therapeutic effect. In this context, supplementing the oleogel with 1,6-hexanediol as the most effective gelation enhancer should drastically reduce the required concentration of TE necessary for gel formation.

**Figure 11:**
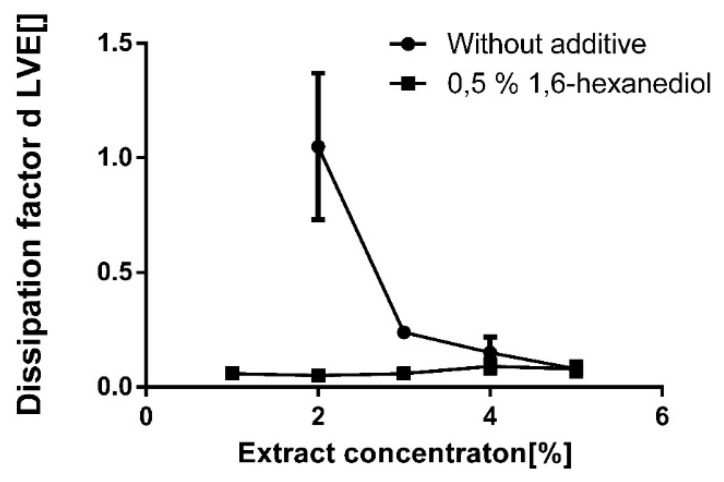
Dissipation factor d with different concentrations of TE with and without 1,6-hexanediol (0.5%); mean ± SD, *n* = 3.

To this end, oleogels with different TE concentrations were produced without and with the addition of a rather low concentration (0.5%) of 1,6-hexanediol (Figure 11).

Without additives, the critical gelation concentration of TE in sunflower oil was 3% as indicated by a dissipation factor in the linear viscoelastic region ≤1. In the presence of 0.5% 1,6-hexanediol, the critical gelation concentration dropped to <1% TE. Even at such low TE concentrations, the resulting oleogels with 1,6-hexanediol are highly elastic as indicated by a dissipation factor <0.1.

## 4. Conclusions

Hydrogen bonds are mainly responsible for the formation of the gel network in oleogels with birch bark dry extract. Additives, which can act as linkers between the extract particles, enhance the formation of a particulate network. The most pronounced effect was observed in diols with terminal hydroxyl groups. 1,6-Hexanediol gave the best results and allowed the formation of an oleogel with a drastically reduced concentration of TE. This effect depends on the concentration of the gelation enhancer. A concentration in the range of 0.5% to 1% was found to be optimal. In contrast, 1,2-diols impaired gel formation by blocking superficial OH groups on the extract particles and preventing them from building a three dimensional particulate network.

## Figures and Tables

**Table 1:** Chemical and physical characteristics of the birch bark dry extract.

Constituent	Amount (*w*/*w* %)	Specific Surface Area (m^2^/g)	Particle Size (μm)
Betulin	80.0	42	5.8
Betulinic acid	3.1		
Oleanolic acid	0.5		
Lupeol	4.3		
Erythrodiol	0.8		
Undisclosed substances	11.3		
